# Red blood cell transfusion in patients with subarachnoid hemorrhage: a multidisciplinary North American survey

**DOI:** 10.1186/cc9977

**Published:** 2011-01-18

**Authors:** Andreas H Kramer, Michael N Diringer, Jose I Suarez, Andrew M Naidech, Loch R Macdonald, Peter D Le Roux

**Affiliations:** 1 Departments of Critical Care Medicine and Clinical Neurosciences, Hotchkiss Brain Institute, Foothills Medical Center, University of Calgary, 1403 29th Street NW, Calgary, AB T2N 2T9, Canada; 2 Department of Neurology and Neurological Surgery, Neurology/Neurosurgery Intensive Care Unit, Washington University School of Medicine, Campus Box 8111, 660 S. Euclid Avenue, St Louis, MO 63110, USA; 3 Department of Neurology, Divisions of Vascular Neurology and Neurocritical Care, Baylor College of Medicine, 6501 Fannin Street, MS: NB320, Houston, TX 77030, USA; 4 Department of Neurology, Feinberg School of Medicine, Northwestern University, 710 N. Lake Shore Drive, Chicago, IL 60611, USA; 5 Division of Neurosurgery, Department of Surgery, St. Michael's Hospital, University of Toronto, 30 Bond Street, Toronto, ON M5B 1W8, Canada; 6 Department of Neurosurgery, University of Pennsylvania, 235 S. 8th Street, Philadelphia, PA 19106, USA

## Abstract

**Introduction:**

Anemia is associated with poor outcomes in patients with aneurysmal subarachnoid hemorrhage (SAH). It remains unclear whether this association can be modified with more aggressive use of red blood cell (RBC) transfusions. The degree to which restrictive thresholds have been adopted in neurocritical care patients remains unknown.

**Methods:**

We performed a survey of North American academic neurointensivists, vascular neurosurgeons and multidisciplinary intensivists who regularly care for patients with SAH to determine hemoglobin (Hb) concentrations which commonly trigger a decision to initiate transfusion. We also assessed minimum and maximum acceptable Hb goals in the context of a clinical trial and how decision-making is influenced by advanced neurological monitoring, clinician characteristics and patient-specific factors.

**Results:**

The survey was sent to 531 clinicians, of whom 282 (53%) responded. In a hypothetical patient with high-grade SAH (WFNS 4), the mean Hb concentration at which clinicians administered RBCs was 8.19 g/dL (95% CI, 8.07 to 8.30 g/dL). Transfusion practices were comparatively more restrictive in patients with low-grade SAH (mean Hb 7.85 g/dL (95% CI, 7.73 to 7.97 g/dL)) (*P *< 0.0001) and more liberal in patients with delayed cerebral ischemia (DCI) (mean Hb 8.58 g/dL (95% CI, 8.45 to 8.72 g/dL)) (*P *< 0.0001). In each setting, there was a broad range of opinions. The majority of respondents expressed a willingness to study a restrictive threshold of ≤8 g/dL (92%) and a liberal goal of ≥10 g/dl (75%); in both cases, the preferred transfusion thresholds were significantly higher for patients with DCI (*P *< 0.0001). Neurosurgeons expressed higher minimum Hb goals than intensivists, especially for patients with high-grade SAH (β = 0.46, *P *= 0.003), and were more likely to administer two rather than one unit of RBCs (56% vs. 19%; *P *< 0.0001). Institutional use of transfusion protocols was associated with more restrictive practices. More senior clinicians preferred higher Hb goals in the context of a clinical trial. Respondents were more likely to transfuse patients with brain tissue oxygen tension values <15 mmHg and lactate-to-pyruvate ratios >40.

**Conclusions:**

There is widespread variation in the use of RBC transfusions in SAH patients. Practices are heavily influenced by the specific dynamic clinical characteristics of patients and may be further modified by clinician specialty and seniority, the use of protocols and advanced neurological monitoring.

## Introduction

The prevention of secondary brain injury is a key paradigm of neurocritical care [1]. Inadequate cerebral oxygen delivery is an important mechanism that may contribute to secondary brain injury. This is particularly true for patients with aneurysmal subarachnoid hemorrhage (SAH), where delayed cerebral ischemia (DCI) and infarction frequently contribute to poor outcomes. When carefully sought, angiographic vasospasm can be observed in about two-thirds of patients during the 2 weeks after aneurysm rupture [2]. Among patients who survive, evidence of acute infarction can be detected in more than 50% of patients with the use of magnetic resonance imaging [3]. In contrast to other neurocritical care conditions, the high risk of delayed ischemia after admission to the hospital provides a unique opportunity to provide neuroprotection prior to additional insults.

Because the concentration of hemoglobin (Hb) is a major determinant of arterial oxygen content, there is a strong therapeutic rationale for the avoidance of anemia in patients with brain injury [4]. Physiological studies have demonstrated improvements in cerebral oxygenation when red blood cell (RBC) transfusions are used to raise Hb levels in anemic SAH patients, particularly when oxygen delivery and cerebral perfusion are reduced [4-7]. Several observational studies have found an association between lower Hb concentrations and poor outcomes [8-10]. Although the correction of anemia is straightforward, the use of allogeneic RBC transfusions to do so has potentially deleterious implications. For example, associations with acute lung injury and nosocomial infections have been described, which could neutralize any physiological advantage [11-13].

Large, multicenter, randomized, controlled trials involving heterogeneous critically ill patients have not found any benefit to the liberal use of RBC transfusions to maintain higher Hb concentrations (>9 to 10 g/dL); however, neurocritical care patients composed only a small subset of the total patient population [14,15]. It is currently unknown to what extent restrictive transfusion thresholds (for example, <7 g/dL) have been adopted in brain-injured patients. A previous international survey suggested that most intensivists still consider a hematocrit level of about 30% to be optimal in SAH patients. However, it does not necessarily follow that clinicians would transfuse liberally to achieve this goal [16]. Furthermore, there are no data indicating how transfusion decisions are guided by multimodal neurological monitoring, which demographic and clinical factors may influence practices and how low (or high) clinicians might allow transfusion thresholds to be in the context of a clinical trial. In view of this uncertainty, we conducted a cross-sectional survey of North American clinicians involved in the decision to administer blood transfusions in critically ill SAH patients.

## Materials and methods

The survey was endorsed by the clinical trials committee of the Neurocritical Care Society. Our sampling frame consisted of neurointensivists, multidisciplinary intensivists who regularly care for SAH patients, and vascular neurosurgeons. We specifically targeted individuals who work at academic institutions with neurocritical care fellowships and/or neurosurgery residency training programs, since these clinicians are the most likely to participate in future clinical trials.

As of March 2010, there were 42 U.S. centers with neurocritical care fellowship programs accredited by the United Council for Neurologic Subspecialties. Through the Society of Neurological Surgeons, we obtained a list of an additional 56 U.S. centers with neurosurgical residency training programs but no accredited neurocritical care fellowship program. Through the Canadian Residency Matching Service website, we identified 12 primarily English-speaking universities with neurosurgery residency programs.

Program directors were contacted to obtain a list of local intensivists and vascular neurosurgeons who care for SAH patients. For centers from which we received no response, we obtained the names and email addresses of relevant individuals from the respective programs' websites.

The survey was self-administered by the respondents, voluntary and submitted online using SurveyMonkey [17]. Individuals were contacted by email, with three subsequent reminders sent at approximately 1-week intervals. No monetary or other incentive was offered for questionnaire completion. Respondents had the option of filling out the survey anonymously.

Survey development was initiated by two investigators (AHK and PDL) on the basis of a PubMed and MEDLINE review of relevant literature [4], with feedback from other experts (MND, AMN and RLM). Themes that were considered important to explore included the following: (1) transfusion thresholds in both low-grade SAH patients (minimal neurological deficits; defined in this study as World Federation of Neurological Surgeons (WFNS) grades 1 to 3) and high-grade SAH patients (presence of stupor or coma; WFNS grade 4 or 5); (2) transfusion thresholds among patients with moderate to severe angiographic or transcranial Doppler (TCD)-defined vasospasm, but no clear symptoms of DCI; (3) transfusion thresholds among patients with angiographic vasospasm and neurological deterioration (that is, DCI); (4) willingness of clinicians to accept transfusion thresholds above or below their usual practices in the setting of a clinical trial; and (5) modification of transfusion thresholds on the basis of information provided by multimodal neurological monitoring.

Most relevant information was collected by presenting an interactive case of a typical patient with aneurysmal SAH who becomes anemic (see appendices in Additional files 1 and 2). Item reduction was accomplished by piloting the survey among three vascular neurosurgeons and four neurointensivists to ensure that it could be completed in approximately 5 minutes and that the most important themes were considered. These preliminary responses were not included as part of the final survey results.

Several subgroup analyses were planned *a priori *to determine how transfusion practices might be modified on the basis of the following factors: (1) geography (United States vs. Canada), (2) base specialty (neurosurgery vs. intensivists), (3) seniority (years in practice), (4) the presence of an institutional transfusion protocol and (5) the use of multimodal neurological monitoring (defined as the use of at least one of the following: brain tissue oxygen tension (P_bt_O_2_) probes, microdialysis catheters, jugular venous oximetry or continuous cerebral blood flow (CBF) monitors).

Statistical analysis was performed using SAS version 9.1 software (SAS Inc., Cary, NC, USA) and MedCalc version 11.3 software (MedCalc, Mariakerke, Belgium). The normality of data was assessed using the Shapiro-Wilk test. Between-group comparisons of continuous data were performed using the Student's *t*-test or the Wilcoxon rank-sum test, depending on the distribution of data. Two-sample paired tests were used where applicable. Clinicians' transfusion thresholds in multiple settings were compared using the Friedman test (a nonparametric approach analogous to repeated measures analysis of variance), and adjustment for multiple comparisons was made using the Bonferroni correction method. Categorical data were assessed using χ^2 ^analysis or Fisher's exact test as appropriate on the basis of the number of responses per cell. Associations between transfusion thresholds and clinician characteristics were explored using generalized linear regression models (Proc GLM in SAS). Multivariable analysis, including all of the variables from our subgroup analysis, was performed using a backward elimination process whereby the least significant variables were discarded one-by-one if *P *> 0.05. Models were assessed for heteroscedasticity using White's test; if present, a heteroscedasticity-consistent standard error was used. We also assessed interactions (effect measure modification) between variables and included the relevant interaction terms in the initial multivariable models if they were statistically significant (*P *< 0.05) in univariate analysis.

## Results

### Demographics

The survey was sent to 531 individuals, from among whom 282 (53%) responded. The response rate was higher in Canada than in the United States (69% vs. 43%; *P *< 0.0001). There were notable cross-border differences in the base specialties of respondents; the majority in the United States were neurologists (55%) and neurosurgeons (23%) compared with internists (37%) and anesthesiologists (27%) in Canada (Table 1).

**Table 1:** Characteristics of survey respondents from the United States and Canada^a^

	United States (*n *= 143)	Canada (*n *= 139)	Total (*n *= 282)	*P *value
Base specialty, %				
Neurology	55%	2%	29%	<0.0001
Neurosurgery	23%	15%	19%	
Anesthesiology	10%	27%	19%	
Internal medicine	7%	37%	22%	
Emergency medicine	4%	6%	5%	
Surgery	1%	12%	6%	
Years of experience, %				
0-3	34%	17%	26%	0.04
4-7	15%	24%	20%	
8-10	10%	14%	11%	
11-15	22%	21%	21%	
16-20	7%	10%	9%	
>20	13%	14%	13%	
Monitoring tools, %				
CT angiography	91%	88%	90%	0.50
CT perfusion	69%	24%	46%	<0.0001
Transcranial Doppler	89%	63%	76%	<0.0001
P_bt_O_2 _probes	34%	6%	21%	<0.0001
Microdialysis catheters	8%	1%	4%	0.005^b^
Continuous CBF probes	14%	0	7%	<0.0001^b^
Jugular bulb oximetry	13%	12%	13%	0.79
MRI perfusion	33%	17%	25%	0.002
None of above	3%	4%	3%	0.75^b^
Use of institutional transfusion protocol, %	55%	50%	52%	0.42

^a^CT, computed tomography; P_bt_O_2_, brain tissue oxygen tension; CBF, cerebral blood flow; MRI, magnetic resonance imaging; ^b^Fisher's exact test.

### Transfusion thresholds in clinical practice

**Figure 1:**
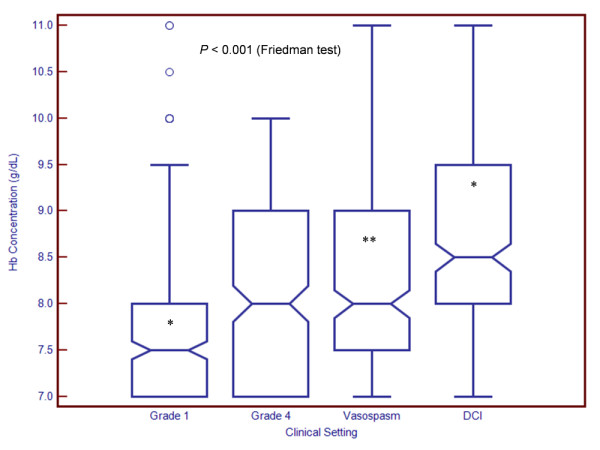
**Hemoglobin (Hb) concentrations at which clinicians transfuse patients with aneurysmal subarachnoid hemorrhage (SAH)**. Boxplots demonstrate median and interquartile range. Circles represent "outside values" (± 1.5 times the interquartile range). Means and 95% confidence intervals are presented in the Results section. DCI, delayed cerebral ischemia; grade refers to World Federation of Neurological Surgeons classification for SAH. *P < 0.0001 in relation to grade 4 SAH (assessed using paired Wilcoxon rank-sum test and Bonferroni correction for multiple comparisons). **P = 0.001 in relation to grade 4 SAH (assessed using paired Wilcoxon rank-sum test and Bonferroni correction for multiple comparisons).

Transfusion thresholds differed significantly, depending on the specific clinical characteristics of the patients (Figure 1) (*P *< 0.001). In a hypothetical patient with WFNS grade 4 SAH (Glasgow Coma Scale (GCS) score of 9, without a focal neurological deficit) and the development of anemia on the third day in the hospital, the mean Hb concentration at which clinicians would choose to administer RBCs was 8.19 g/dL (95% confidence interval (95% CI), 8.07 to 8.30; medians and interquartile ranges (IQRs) are presented in Figure 1). However, opinions varied widely from as low as 7 g/dL (26%) to as high as 10 g/dL (13%).

Transfusion practices were more restrictive in a patient with WFNS grade 1 SAH (GCS score 15) (mean Hb, 7.85 g/dL; 95% CI, 7.73 to 7.97 (*P *< 0.0001 compared with grade 4 SAH)). In contrast, in a patient with evidence of moderate to severe TCD vasospasm (middle cerebral artery flow velocities 180 to 205 cm/second, Lindegaard ratio 5 or 6) on the sixth day in the hospital, without any coinciding neurological deterioration, the mean transfusion threshold rose to 8.35 g/dL (95% CI, 8.22 to 8.48; *P *= 0.001 compared with the same patient on day 3 without TCD vasospasm). When there were both angiographic vasospasm and concomitant observable neurological deterioration (that is, DCI), the mean threshold was even higher at 8.58 g/dL (95% CI, 8.45 to 8.72; *P *< 0.0001 compared with the same patient on day 6 with only TCD vasospasm). For each clinical scenario, there was a wide range of responses (Figure 1).

For patients with Hb concentrations slightly below (<1 g/dL) clinicians' usual transfusion threshold, most respondents (74%) initially administered 1 U of RBCs, while a minority (26%) routinely gave 2 U of RBCs. The proportion that administered 2 U of RBCs was larger in the United States than in Canada (34% vs. 17%; *P *= 0.002) and among neurosurgeons compared with intensivists (56% vs. 19%; *P *< 0.0001).

### Transfusion thresholds in a randomized, controlled trial

**Figure 2:**
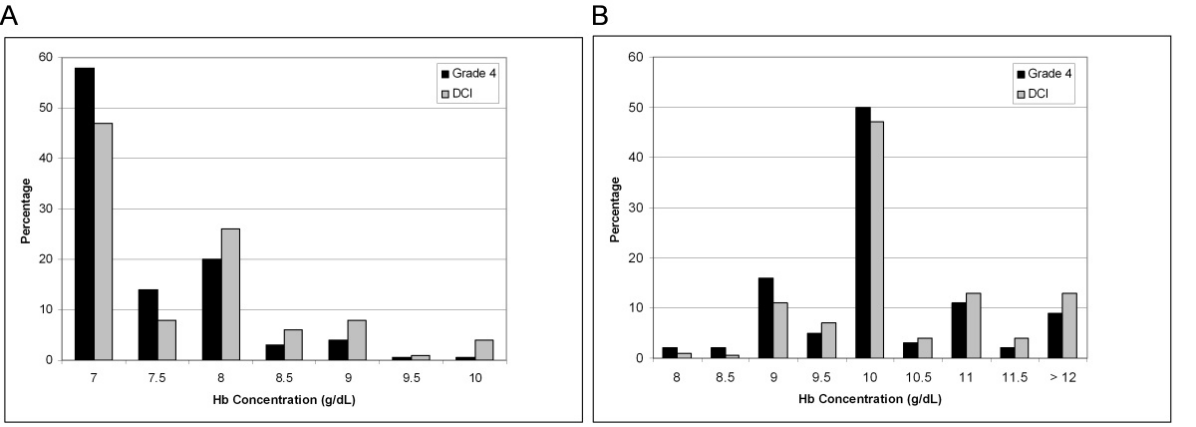
**Minimum and maximum hemoglobin (Hb) concentrations which clinicians consider acceptable thresholds for a randomized trial**. **(A) **Minimum acceptable transfusion threshold. **(B) **Maximum acceptable transfusion threshold. DCI, delayed cerebral ischemia. "Grade" refers to World Federation of Neurological Surgeons classification.

In the patient with grade 4 SAH, 63% of respondents expressed a willingness to accept a Hb threshold lower than their own in a clinical trial. When clinicians with the most restrictive threshold (7 g/dL) were excluded, the proportion rose to 84%. More than 70% of respondents thought it was ethically acceptable to randomize patients to a transfusion trigger as low as 7 or 7.5 g/dL (Figure 2A). Similarly, 94% of respondents were willing to accept a Hb threshold higher than their own in a study, in most cases ≥10 g/dL (Figure 2B).

Acceptable lower transfusion thresholds were influenced by the presence or absence of DCI (mean acceptable threshold with DCI, 7.69 g/dL (median, 7.5; IQR, 7.0 to 8.0); mean acceptable threshold without DCI, 7.41 g/dL (median, 7.0; IQR, 7.0 to 8.0); *P *< 0.0001). However, even in patients with DCI, 63% of respondents expressed their willingness to study a Hb threshold lower than their own (84% when those with a threshold of 7 g/dL were excluded). More than half supported allocating patients to a transfusion trigger of 7 or 7.5 g/dL (Figure 2A). Ninety percent of respondents were willing to study a Hb target higher than their own. The majority favored an upper target of 10 g/dL, but a sizable proportion were willing to increase transfusion to levels exceeding 11 g/dL (Figure 2B). The mean upper acceptable Hb target was greater in patients who develop DCI (mean, 10.31 g/dL (median, 10.0; IQR, 10.0 to 11.0) vs. mean 10.11 g/dL (median, 10.0; IQR, 10.0 to 10.5); *P *< 0.0001).

### Clinician characteristics influencing transfusion practices: subgroup analysis

**Figure 3:**
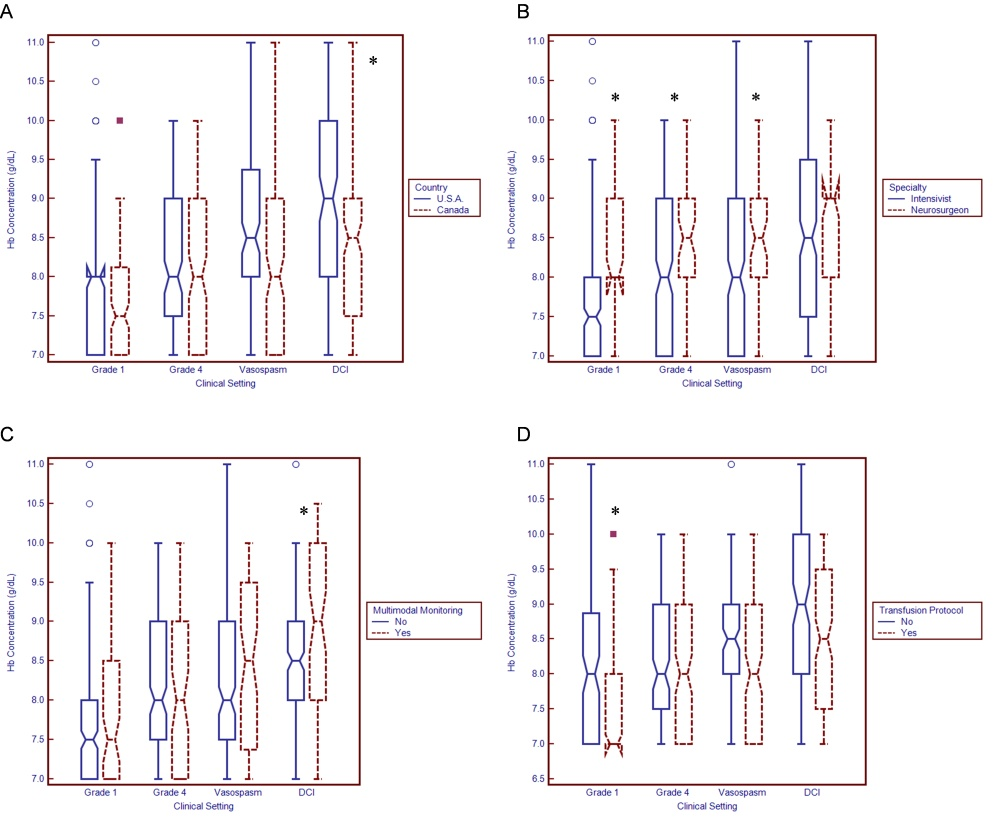
**Relationship between respondent characteristics and transfusion thresholds**. Boxplots demonstrate the median and interquartile range. Circles represent "outside values" (± 1.5 times the interquartile range). Boxes represent "far out values" (± 3 times the interquartile range). **(A) **Country. **(B) **Specialty. The term "intensivist" refers both to individuals who practice exclusively as neurointensivists and to multidisciplinary intensivists who regularly care for patients with subarachnoid hemorrhage. **(C) **Use of multimodal neurological monitoring. **(D) **Use of transfusion protocol. DCI, delayed cerebral ischemia; Hb, hemoglobin. "Grade" refers to World Federation of Neurological Surgeons classification. *P < 0.05 using the Wilcoxon rank-sum test.

**Figure 4:**
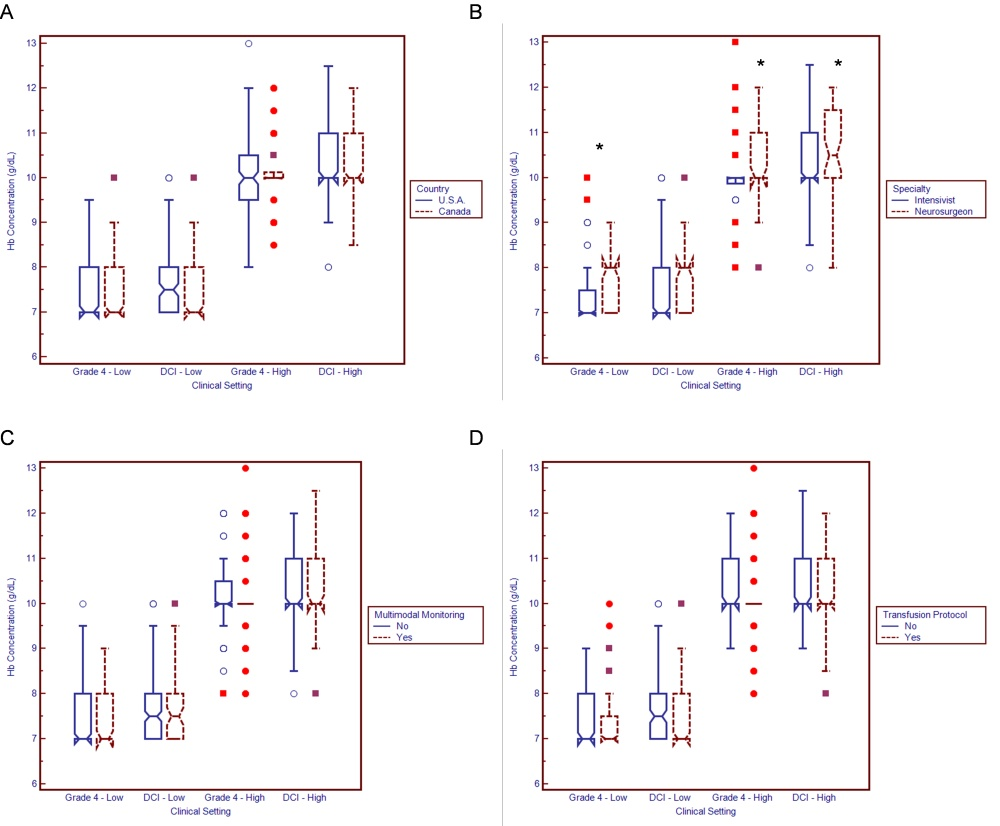
**Relationship between respondent characteristics and acceptable transfusion thresholds in the setting of a randomized, controlled trial**. Boxplots demonstrate median and interquartile range. Circles represent "outside values" (± 1.5 times the interquartile range). Boxes represent "far out values" (± 3 times the interquartile range). **(A) **Country. **(B) **Specialty. The term "intensivist" refers both to individuals who practice exclusively as neurointensivists and to multidisciplinary intensivists who regularly care for patients with subarachnoid hemorrhage. **(C) **Use of multimodal neurological monitoring. **(D) **Use of transfusion protocol. DCI, delayed cerebral ischemia; Hb, haemoglobin. "Grade" refers to World Federation of Neurological Surgeons classification. *P < 0.05 using the Wilcoxon rank-sum test.

U.S. clinicians consistently reported transfusing at higher Hb concentrations than Canadian clinicians (Figure 3A). However, this difference reached statistical significance only for patients with DCI (mean Hb level among U.S. clinicians, 8.74 g/dL (95% CI, 8.55 to 8.92); mean Hb level among clinicians in Canada, 8.44 g/dL (95% CI, 8.25 to 8.63); *P *= 0.03). There were no major differences in the maximum and minimum Hb concentrations that clinicians from either country would consider acceptable in a randomized, controlled trial (Figure 4A).

Neurosurgeons reported more liberal transfusion thresholds than did intensivists (Figure 3B). Differences were statistically significant for patients with grade 4 SAH, grade 1 SAH and TCD vasospasm. Neurosurgeons were also less willing than intensivists to accept very low Hb concentrations (7 to 7.5 g/dL) in the setting of a randomized, controlled trial and were more willing to transfuse to relatively high Hb targets (10 to 11.5 g/dL) (Figure 4B).

Clinicians who routinely use multimodal neurological monitoring in SAH patients report targeting higher Hb concentrations, especially in patients with DCI (mean Hb with multimodal monitoring, 8.82 g/dL (95% CI, 8.55 to 0.10); mean Hb without monitoring, 8.50 g/dL (95% CI, 8.35 to 8.66); *P *= 0.04) (Figure 3C). Appendix 2 in Additional file 2 shows specifically how the use of P_bt_O_2 _and microdialysis monitoring may modify practices. The use of institutional transfusion protocols was associated with more restrictive thresholds, especially in grade 1 SAH patients (mean Hb with transfusion protocol, 7.70 g/dL (95% CI, 7.54 to 7.87); mean Hb without protocol, 7.98 g/dL (95% CI, 7.80 to 8.16); *P *= 0.01) (Figure 3D).

There were no significant associations between clinician experience (years in practice) and conventional transfusion practices in any of the clinical settings. However, more experienced respondents were less willing to accept lower Hb thresholds in the restrictive arm of a randomized, controlled trial (grade 4 SAH, β = 0.01, *P *= 0.009; DCI, β = 0.02, *P *= 0.01). In the liberal transfusion group, more experienced respondents reported that they would be willing to transfuse to higher Hb targets (grade 4 SAH, β = 0.02, *P *= 0.04; DCI, β = 0.02, *P *= 0.01).

### Clinician characteristics influencing transfusion practices: multivariable analysis

Using multivariable analysis, several independent predictors of transfusion practices were identified (Table 2). Hemoglobin thresholds were more liberal among neurosurgeons than among intensivists (grade 4 SAH, β = 0.46, *P *= 0.003; TCD vasospasm, β = 0.31, *P *= 0.04) and more restrictive among clinicians who use transfusion protocols (grade 1 SAH, β = -0.42, *P *= 0.0008). In grade 1 SAH patients, we also found significant effect measure modification between the use of a protocol and neurosurgical specialty (β = 0.88, *P *< 0.0001). For example, although the use of a protocol generally predicted more restrictive practices, the opposite was true among neurosurgeons (mean transfusion threshold with protocol, 8.43 g/dL (95% CI, 8.00 to 8.86); mean threshold without protocol, 8.18 g/dL (95% CI, 7.81 to 8.55)). In patients with DCI, the use of multimodal neurological monitoring remained independently associated with a more liberal transfusion threshold (β = 0.32, *P *= 0.04).

**Table 2:** Multivariable analysis assessing associations between respondent characteristics and transfusion thresholds in clinical practice^a^

Clinical setting value	Predictors remaining in final model	Estimate (β)	*P*
WFNS grade 4 (day 3)	Specialty (neurosurgery)	0.46	0.003
WFNS grade 1 (day 3)	Transfusion protocol	-0.42	0.0008
	Transfusion protocol^b^ Specialty (neurosurgery)^c^	0.88	<0.0001
TCD vasospasm (day 6)	Specialty (neurosurgery)	0.31	0.04^d^
DCI (day 7)	Multimodal neurological monitoring	0.32	0.04

^a^Multivariable analysis was performed using generalized linear models with stepwise backward elimination of the least significant variable where *P *> 0.05. Initial models included country (United States vs. Canada), specialty (neurosurgery vs. critical care), multimodal monitoring (yes vs. no), use of a transfusion protocol (yes vs. no) and years in practice (continuous variable). All interactions were assessed, and those for which *P *< 0.05 in univariate analysis were incorporated into initial multivariable models. WFNS, World Federation of Neurological Surgeons scale; TCD, transcranial Doppler; DCI, delayed cerebral ischemia. ^b^Neurosurgical specialty significantly modified practices among clinicians who use a protocol (see Results section for details); ^c^Years in practice significantly modified practices among neurosurgeons (see Results section for details); ^d^White's heteroscedasticity-specific standard error.

In the context of a randomized, controlled trial, neurosurgical specialty and increased clinician seniority were associated with less willingness to accept very restrictive transfusion thresholds (Tables 2 and 3). In the liberal transfusion arm, we found significant effect measure modification between neurosurgical specialty and years in practice. The highest Hb targets were generally found among neurosurgeons with a greater degree of experience. For example, among neurosurgeons who had been in practice for more than 10 years, the mean highest acceptable Hb goal was 10.60 g/dL (95% CI, 10.13 to 11.07) compared with 10.04 g/dL (95% CI, 9.62 to 10.45) among neurosurgeons in practice for fewer years and 10.22 g/dL (95% CI, 10.03 to 10.41) among intensivists in practice for more than 10 years.

**Table 3:** Multivariable analysis assessing associations between respondent characteristics and transfusion thresholds in the context of a randomized, controlled trial^a^

Clinical setting	Predictors remaining in final model	Estimate (β)	*P *value
WFNS grade 4			
(lowest acceptable Hb)	Specialty (neurosurgery)	0.37	<0.0001
	Years in practice	0.01	0.009
WFNS grade 4			
(highest acceptable Hb)	Specialty (neurosurgery)^b^ Years in practice^c,d^	0.03	0.01
DCI			
(lowest acceptable Hb)	Years in practice	0.02	0.01
DCI			
(highest acceptable Hb)	Transfusion protocol	-0.35	0.003
	Years in practice	0.02	0.007
	Transfusion protocol^b^ Specialty (neurosurgery)^b^	0.66	0.002

^a^Multivariable analysis was performed using generalized linear models with stepwise backward elimination of the least significant variable where *P *> 0.05. Initial models included country (United States vs. Canada), specialty (neurosurgery vs. critical care), multimodal monitoring (yes vs. no), use of a transfusion protocol (yes vs. no) and years in practice (continuous variable). All interactions were assessed, and those for which *P *< 0.05 in univariate analysis were incorporated into initial multivariable models. WFNS, World Federation of Neurological Surgeons scale; Hb, hemoglobin concentration; DCI, delayed cerebral ischemia. ^b^Neurosurgical specialty significantly modified practices among clinicians who use a protocol (see Results section for details); ^c^White's heteroscedasticity-specific standard error; ^d^Years in practice significantly modified practices among neurosurgeons (see Results section for details).

Clinicians using transfusion protocols were less willing to target higher Hb goals in patients with DCI (β = -0.35, *P *= 0.003). However, this effect was modified by clinician specialty; for example, the mean highest acceptable Hb target was 10.70 g/dL (95% CI, 10.26 to 11.15) among neurosurgeons using a protocol, but only 10.57 g/dL (95% CI, 10.14 to 11.00) when no protocol was used.

## Discussion

Our findings describe current RBC transfusion practices in patients with SAH at North American academic centers. We observed variations in the Hb concentrations which trigger a decision to initiate transfusion, distributed over a numerically modest but clinically significant range of 7 to 11 g/dL (Figure 1). Although a threshold of 7 g/dL is widely advocated for general critical care patients, tolerance for such a low Hb level is less common in SAH patients. The variability in clinicians' practices provides a strong impetus for a definitive randomized, controlled trial.

Many clinicians do not practice with a fixed Hb threshold. Instead, the decision to initiate transfusion varies on the basis of the clinical status of the patient. Survey respondents were more likely to initiate transfusion in patients with high-grade rather than low-grade SAH. This practice suggests that clinicians believe anemia to be potentially more harmful among patients with a greater degree of brain injury. Clinicians are even more likely to initiate transfusion if patients develop cerebral vasospasm, especially if there is concomitant neurological deterioration (that is, DCI). This observation indicates that most clinicians do not consider marked hemodilution to be an appropriate method of treating vasospasm and DCI. Indeed, although hemodilution increases CBF, this practice may compromise oxygen delivery [18,19]. In some SAH patients, there may be additional systemic factors (for example, neurogenic cardiac dysfunction or known coronary artery disease) which may influence the decision to initiate transfusion; these factors were not incorporated into this survey.

The stated willingness of most clinicians to modify their transfusion practices in the context of a randomized, controlled trial further demonstrates equipoise. Almost three-fourths of respondents considered it reasonable to randomize a patient with grade 4 SAH to a transfusion threshold of 7 or 7.5 g/dL. However, clinicians were less willing to accept such a low Hb when patients develop DCI. The vast majority also thought it was acceptable to target a Hb concentration of >10 g/dL as part of a liberal transfusion strategy. Among patients with DCI, a notable proportion of clinicians were willing to target even higher Hb levels. These findings suggest that a comparison of two fixed Hb thresholds may not represent the most relevant approach to study in a randomized, controlled trial. Indeed, it has been pointed out that there may be unintended harmful consequences in studies that use fixed treatment protocols for therapies that are more often titrated in day-to-day practice [20]. An alternative approach is to use adaptive trial designs in which therapy titration is permitted on the basis of prospective rules. For example, the upper and lower transfusion triggers could be adjusted on the basis of the presence or absence of DCI and radiographic evidence of vasospasm.

Our findings suggest that vascular neurosurgeons are less tolerant than intensivists regarding Hb reductions. Neurosurgeons were also more likely to administer 2 U rather than 1 U of RBCs when Hb levels dropped below their usual transfusion thresholds. These findings are consistent with a previous survey which reported that U.S. neurosurgeons are more likely than trauma surgeons or intensivists to target Hb concentrations of at least 10 g/dL in patients with severe traumatic brain injury [21].

We identified additional factors which may influence the transfusion decision. Although differences were small, clinicians who reported using a transfusion protocol generally appeared to be slightly more restrictive in their use of RBCs. This was not true for vascular neurosurgeons; however, our data do not allow us to determine whether this apparent discrepancy is due to variations in protocols, lack of compliance or chance. The preferred transfusion thresholds in a randomized trial would be higher for clinicians with a greater degree of seniority. The reasons for this observation are also unclear. Possibilities could include less familiarity with published literature advocating restrictive transfusion strategies, increased skepticism regarding the applicability of such studies to SAH patients or greater reluctance to adapt practices. These observations, together with interdisciplinary and international differences in transfusion preferences, should be taken into consideration in the planning of future studies. This will help maximize clinician buy-in and ensure that study results are widely generalizable.

This survey is the first to assess how transfusion practices are influenced by advanced neurological monitoring. We found that clinicians who use invasive, multimodal neurological monitoring may be more liberal in their use of transfusions, especially among patients with DCI. To keep the survey brief, we restricted further questioning to the use of P_bt_O_2 _probes and microdialysis catheters (see Appendix 2 in Additional file 2). The majority of clinicians are more likely to transfuse when P_bt_O_2 _values fall below 15 mmHg. A considerably smaller proportion are more likely to initiate transfusion when the P_bt_O_2 _level is 15 to 20 mmHg. It is important to point out that transfusion is usually considered as a method to raise P_bt_O_2 _only if other strategies (for example, optimizing cerebral perfusion pressure and partial pressure of oxygen) have failed. A definitive, "critical" P_bt_O_2 _threshold value has never been identified with certainty. On the basis of associations with poor outcomes, levels of 10 to 20 mmHg have been advocated both in patients with severe traumatic brain injury and in patients with SAH [22-24]. Evidence of ischemia found by using positron emission tomography has been demonstrated at a P_bt_O_2 _threshold of approximately 14 mmHg [25]. An ongoing National Institutes of Health-sponsored phase II clinical trial in traumatic brain injury patients uses a P_bt_O_2 _threshold of 20 mmHg to initiate therapy [26]. The clinical significance of an elevated lactate-to-pyruvate ratio (LPR) is less clear to clinicians; only one-third of respondents indicated that an LPR value greater than 40 would influence them to initiate transfusion. However, experience with microdialysis in patients with SAH is limited in North America (Table 1). Among clinicians who report regular use of microdialysis, a LPR threshold of about 35 to 40 appears to be considered critical. A high LPR has been shown to be predictive of poor outcome after SAH [27]. However, pronounced LPR elevations may occur in the absence of ischemia [28] and may not be modified by the administration of RBCs [29].

Survey validity is enhanced by a high response rate. To maximize responses, we corresponded with program directors prior to initiation of the survey, sent three reminder emails to potential respondents and deliberately kept the questionnaire short. The survey was case-based, with interactive scenarios designed to reflect typical clinical practice. Our response rate (53%) is relatively consistent with that of other published surveys of physicians [30,31]. However, as with most surveys, it is impossible for us to determine whether there were systematic differences in transfusion practices between responders and nonresponders. In addition, there may be differences between what clinicians perceive that they do and how they actually practice. Because of a higher response rate, we can be more confident of the validity of our findings among Canadian clinicians than among U.S. clinicians.

Although our sampling frame was selected specifically to target clinicians most influential in the care of patients with SAH, we may not have surveyed all potential decision makers; in particular, we did not include responses from residents or nurse practitioners. Since the survey was performed without any funding, we did not provide a monetary (or other) incentive and chose to perform only an Internet-based rather than a postal questionnaire. There are some data to suggest that response rates are higher in postal surveys [32]. On the other hand, our response rate, especially from Canadian intensivists and neurosurgeons, compares favorably with what has been reported elsewhere [30-32]. Because most respondents completed the survey anonymously, we could not record at which particular center they work. Thus, it is possible that our results could have been influenced by variations in the number of responders per center, and it is conceivable that this could have led us to underestimate the degree of variability in transfusion practices. Finally, it remains unclear to what degree our findings reflect current practices in other regions of the world.

## Conclusions

There is widespread variation in practices regarding the use of RBC transfusions in the management of SAH patients at North American academic medical centers. Equipoise is further demonstrated by the willingness of clinicians to compare relatively divergent Hb transfusion thresholds in the context of a randomized clinical trial. Transfusion practices are heavily influenced by the specific dynamic clinical characteristics of patients and may be further modified by clinician specialty, the use of protocols and clinicians' years in practice.

## Key messages

• There is widespread practice variation in the use of RBC transfusions among North American clinicians caring for critically ill patients with aneurysmal SAH. Most clinicians do not use an Hb transfusion trigger of 7 g/dL and are willing to modify their usual practices in the context of a randomized, controlled trial.

• Clinicians target higher Hb goals among patients with higher-grade SAH and in the presence of cerebral vasospasm or DCI. Thus, comparison of "fixed" Hb thresholds applied regardless of specific clinical circumstances may not represent the optimal approach in future clinical trials assessing "liberal" versus "restrictive" transfusion practices.

• There are significant interdisciplinary differences in clinicians' transfusion practices. Vascular neurosurgeons appear to be more aggressive than intensivists in their use of RBC transfusions. International differences between American and Canadian practices were also observed.

• Most clinicians are more likely to initiate transfusion in patients if P_bt_O_2 _is <15 mmHg. There is more uncertainty when P_bt_O_2 _is 15 to 20 mmHg and with information derived from cerebral microdialysis (lactate-to-pyruvate ratio).

## Abbreviations

CBF: cerebral blood flow; DCI: delayed cerebral ischemia; GCS: Glasgow Coma Scale; Hb: hemoglobin; IQR: interquartile range; LPR: lactate-to-pyruvate ratio; MRI: magnetic resonance imaging; P_bt_O_2_: brain tissue oxygen tension; RBC: red blood cell; SAH: subarachnoid hemorrhage; TCD: transcranial Doppler; WFNS: World Federation of Neurological Surgeons score.

## Competing interests

The authors declare that they have no competing interests.

## Authors' contributions

AHK and PL conceived, designed and carried out the survey. They were also responsible for the analysis and interpretation of the data as well as the drafting and revision of the manuscript. JIS, AMN and RLM assisted in designing the survey, interpreting the data and revising the manuscript. All authors approved the final manuscript.

## Supplementary Material

Additional file 1 **Appendix 1**. Copy of online survey used to collect data for this study (Canadian version). Click here for file

Additional file 2 **Appendix 2**. Modification of transfusion practices on the basis of information provided by P_bt_O_2 _and microdialysis (lactate-to-pyruvate ratio) monitoring. Click here for file
