# Supplementary material for: Red blood cell transfusion in patients with subarachnoid hemorrhage: a multidisciplinary North American survey
Source: Crit Care. 2011 Jan 18;15(1):R30. doi: 10.1186/cc9977 (PMC3222066; doi:10.1186/cc9977)
Supplement: Additional file 1 — Appendix 1. Copy of online survey used to collect data for this study (Canadian version). [file cc9977-S1.PDF]

Transfusion Thresholds In Subarachnoid Hemorrhage - Canada

1. Background Information

1. Please provide your name (confidential and optional). We require this information only to know whether to send a reminder e-mail. If you would prefer to leave this blank, we will send you three reminders (at one week intervals).

2. What is your base specialty (even if you function exclusively as an intensivist, please check the most appropriate box based on your background)?:

- Neurosurgery
- Neurology
- Anesthesia
- Internal Medicine +/- Subspecialty
- Emergency Medicine
- Surgery (Other Than Neurosurgery)

3. For how many years have you been in practice (since completing residency/fellowship training; please check one)?

- 0-3
- 4-7
- 8-10
- 11-15
- 16-20
- > 20

4. Which of the following monitoring modalities, if any, do you commonly use in the care of patients with SAH?

- Transcranial Doppler
- Brain tissue oxygen tension
- Microdialysis
- Continuous CBF monitor
- Jugular venous oximetry
- CT angiography
- CT perfusion studies
- MR perfusion studies
- None of these

5. Which of the following blood conservation strategies do you use in the care of SAH patients:

	Always	Frequently	Rarely	Never	I don't know
Hematopoiesis-stimulating medications (e.g. erythropoietin, darbopoietin)					
Small volume pediatric-sized collection vials					
Iron supplementation (assuming not Fe-deficient)					
Closed arterial system allowing re-injection of "wasted" blood after sampling via arterial line					
Protocol for blood transfusion thresholds					

Transfusion Thresholds In Subarachnoid Hemorrhage - Canada

2. Transfusion Thresholds - Part 1

1. A 56 year old woman with a past history of smoking and hypertension is admitted to your center with a World Federation of Neurological Surgeons grade 4 subarachnoid hemorrhage (SAH).

On examination, she opens her eyes only to painful stimulation, makes moaning sounds, and consistently localizes to pain with both upper extremities. No focal neurological deficit is noted. Her CT scan reveals diffuse subarachnoid blood, with some intraventricular hemorrhage and mild hydrocephalus. A CT angiogram reveals an anterior communicating artery aneurysm, 4 mm in diameter. Her neurological assessment improves slightly with placement of a ventriculostomy (intermittently follows simple commands). Her ICP is not elevated, and the drain is left open 15 cm above the external auditory meatus.

On day 2 of hospitalization, she undergoes craniotomy with surgical clip ligation of the aneurysm. Post-operatively, her neurological status is unchanged, and she is extubated. On day 3, routine morning lab work reveals her hemoglobin concentration to be 98 g/L.

What Hb threshold do you believe would be most appropriate for transfusing this patient?

- < 70 g/dL
- < 75 g/L
- < 80 g/L
- < 85 g/L
- < 90 g/L
- < 95 g/L
- < 100 g/L
- < 105 g/L
- < 110 g/L
- < 115 g/L
- < 120 g/L

2. Assuming her Hb concentration is slightly (< 10 g/L) below your usual threshold, how many units of red blood cells would you administer?

- 1
- 2

3. If you were asked to enroll this patient in a randomized controlled trial evaluating transfusion thresholds, what would be the highest Hb concentration you believe would be ethically justifiable to target in a "liberal" transfusion arm (even if you never actually target such a high Hb in your own practice?)

- > 75 g/L
- > 80 g/L
- > 85 g/L
- > 90 g/L
- > 95 g/L
- > 100 g/L
- > 105 g/L
- > 110 g/L
- > 115 g/L
- > 120 g/L
- > 125 g/L
- > 130 g/L

4. Similarly, what would be the lowest Hb concentration you would accept for this patient in the "restrictive" transfusion arm of a clinical trial (even if it is lower than your preferred threshold)?

- < 70 g/L
- < 75 g/L
- < 80 g/L
- < 85 g/L
- < 90 g/L
- < 95 g/L
- < 100 g/L
- < 105 g/L
- < 110 g/L

5. Even if you do not normally use PbtO2 monitoring, please provide a response. Would you be more likely to transfuse this patient if:

	Yes	No	Not sure
A. Brain tissue oxygen tension was < 15 mmHg?			
B. Brain tissue oxygen tension was 15.1-20 mmHg?			
C. Brain tissue oxygen tension was 20.1-25 mmHg?			

6. Even if you do not normally use microdialysis, please provide a response. Would you be more likely to transfuse this patient if:

	Yes	No	Not sure
A. Microdialysis lactate:pyruvate ratio was > 40?			
B. Microdialysis lactate:pyruvate ratio was 35.1-39.9?			
C. Microdialysis lactate:pyruvate ratio was 30.1-35?			
D. Microdialysis lactate:pyruvate ratio was 25.1-30?			

7. If you were presented with a similar scenario to the above case, but the patient instead had no neurological deficit (Glasgow Coma Scale 15, World Federation of Neurological Surgeons Scale 1), would this modify your approach? What would be the appropriate transfusion threshold?

- < 70 g/L
- < 75 g/L
- < 80 g/L
- < 85 g/L
- < 90 g/L
- < 95 g/L
- < 100 g/L
- < 105 g/L
- < 110 g/L
- < 115 g/L
- < 120 g/L

3. Transfusion Thresholds - Part 2

1. Returning to our original case, it is now day 6 following SAH. The patient's neurological status has clearly improved since the immediate post-operative period. She remains lethargic, but opens her eyes spontaneously, answers "yes" and "no" to simple questions and follows one step commands. However, you note that her middle (MCA) and anterior cerebral artery (ACA) transcranial Doppler velocities have gradually increased over the past 72 hours. Her mean MCA flow velocities are 180 cm/sec on the right and 205 cm/sec on the left. The Lindegaard ratios are 5.1 and 6.0, respectively (consistent with at least moderate vasospasm). After receiving a transfusion earlier in the admission, her Hb has again drifted down to 95 g/L.

At this time, how low would her Hb have to be for you to transfuse?

- < 70 g/L
- < 75 g/L
- < 80 g/L
- < 85 g/L
- < 90 g/L
- < 95 g/L
- < 100 g/L
- < 105 g/L
- < 110 g/L
- < 115 g/L
- < 120 g/L

2. The following morning, her TCD velocities are only slightly higher, but she is more difficult to rouse. There is no verbal response, and she no longer follows commands. She requires endotracheal intubation to facilitate a repeat CT scan and concomitant CT angiography. This reveals diffuse, moderate-severe vasospasm (> 50% narrowing relative to initial CT angiogram) in her middle and anterior cerebral arteries.

At this time, how low would her Hb have to be for you to transfuse?

- < 70 g/L
- < 75 g/L
- < 80 g/L
- < 85 g/L
- < 90 g/L
- < 95 g/L
- < 100 g/L
- < 105 g/L
- < 110 g/L
- < 115 g/L
- < 120 g/L

3. If you were asked to enroll this patient (in question #2) into a randomized controlled trial evaluating transfusion thresholds, what would be the highest Hb concentration you believe would be ethically justifiable to target in a "liberal" transfusion arm (even if you never actually target such a high Hb in your own practice)?

- > 80 g/L
- > 85 g/L
- > 90 g/L
- > 95 g/L
- > 100 g/L
- > 105 g/L
- > 110 g/L
- > 115 g/L
- > 120 g/L
- > 125 g/L
- > 130 g/L

4. Similarly, what would be the lowest Hb concentration you would accept for this patient in the "restrictive" transfusion arm of a clinical trial (even if it is lower than your preferred threshold)?

- < 70 g/L
- < 75 g/L
- < 80 g/L
- < 85 g/L
- < 90 g/L
- < 95 g/L
- < 100 g/L
- < 105 g/L
- < 110 g/L
- < 115 g/L
- < 120 g/L
