# Supplementary material for: Red blood cell transfusion in patients with subarachnoid hemorrhage: a multidisciplinary North American survey
Source: Crit Care. 2011 Jan 18;15(1):R30. doi: 10.1186/cc9977 (PMC3222066; doi:10.1186/cc9977)
Supplement: Additional file 2 — Appendix 2. Modification of transfusion practices on the basis of information provided by PbtO2 and microdialysis (lactate-to-pyruvate ratio) monitoring. [file cc9977-S2.DOC]

Appendix 2. Modification of transfusion practices based on information provided by P_bt_O_2_ and microdialysis (lactate:pyruvate) monitoring

A. P_bt_O_2_

B. Microdialysis

Abbreviations: P_bt_O_2_ = brain tissue oxygen tension

Approximately two-thirds of clinicians responded that they are more likely to transfuse if P_bt_O_2_ levels fall below 15 mmHg in non-infarcted brain tissue, compared with only 22% when the P_bt_O_2_ is 15-20 mmHg and almost none when the P_bt_O_2_ is 20-25 mmHg. For clinicians who commonly use P_bt_O_2_ monitors, these proportions were 85%, 41% and 0%, respectively.

There was less certainty regarding use of information derived from microdialysis catheters. About one-third of respondents indicated that they are more likely to transfuse when the LPR is greater than 40. For clinicians who commonly use microdialysis, the proportions that are more likely to transfuse with a LPR of > 40, 35-40, or 25-35 were 90%, 60% and 0%, respectively.
